# Physiological Response to the COVID-19 Vaccine: Insights From a Prospective, Randomized, Single-Blinded, Crossover Trial

**DOI:** 10.2196/51120

**Published:** 2024-07-31

**Authors:** Andjela Markovic, Vladimir Kovacevic, Timo B Brakenhoff, Duco Veen, Paul Klaver, Marianna Mitratza, George S Downward, Diederick E Grobbee, Maureen Cronin, Brianna M Goodale, COVID-19 Remote Early Detection (COVID-RED) consortium

**Affiliations:** 1 Department of Psychology University of Fribourg Fribourg Switzerland; 2 Department of Social Neuroscience and Social Psychology Institute of Psychology University of Bern Bern Switzerland; 3 Department of Pulmonology University Hospital Zurich Zurich Switzerland; 4 Ava Aktiengesellschaft (AG) Zurich Switzerland; 5 The Institute for Artificial Intelligence Research and Development of Serbia Belgrade Serbia; 6 Julius Clinical Zeist Netherlands; 7 Department of Methodology & Statistics Utrecht University Utrecht Netherlands; 8 Optentia Research Programme North-West University Potchefstroom South Africa; 9 Julius Global Health Julius Center for Health Sciences and Primary Care University Medical Center Utrecht Netherlands; 10 See Acknowledgments

**Keywords:** wearable technology, biosignals, digital health, SARS-CoV-2, vaccine reactogenicity, menstrual cycle, vaccine, wearables, sex, development, implementation, medical device, breathing rate, heart rate, biological mechanism, immune response, vaccination

## Abstract

**Background:**

Rapid development and implementation of vaccines constituted a crucial step in containing the COVID-19 pandemic. A comprehensive understanding of physiological responses to these vaccines is important to build trust in medicine.

**Objective:**

This study aims to investigate temporal dynamics before and after COVID-19 vaccination in 4 physiological parameters as well as the duration of menstrual cycle phases.

**Methods:**

In a prospective trial, 17,825 adults in the Netherlands wore a medical device on their wrist for up to 9 months. The device recorded their physiological signals and synchronized with a complementary smartphone app. By means of multilevel quadratic regression, we examined changes in wearable-recorded breathing rate, wrist skin temperature, heart rate, heart rate variability, and objectively assessed the duration of menstrual cycle phases in menstruating participants to assess the effects of COVID-19 vaccination.

**Results:**

The recorded physiological signals demonstrated short-term increases in breathing rate and heart rate after COVID-19 vaccination followed by a prompt rebound to baseline levels likely reflecting biological mechanisms accompanying the immune response to vaccination. No sex differences were evident in the measured physiological responses. In menstruating participants, we found a 0.8% decrease in the duration of the menstrual phase following vaccination.

**Conclusions:**

The observed short-term changes suggest that COVID-19 vaccines are not associated with long-term biophysical issues. Taken together, our work provides valuable insights into continuous fluctuations of physiological responses to vaccination and highlights the importance of digital solutions in health care.

**International Registered Report Identifier (IRRID):**

RR2-10.1186/s13063-021-05241-5

## Introduction

In December 2020, the first COVID-19 vaccine worldwide was made available to the public under emergency use authorization to contain the COVID-19 pandemic [1]. Subsequently, national COVID-19 vaccination programs were rolled out across the globe. COVID-19 vaccines were shown to significantly reduce the risk of infection [2] as well as reinfection [3]. Furthermore, it was demonstrated that hospitalization due to COVID-19 was less likely in vaccinated individuals, as was a severe disease course with progression to mechanical ventilation or death [4].

Similar to routine vaccines, mild side effects have been reported for COVID-19 vaccines. These side effects most commonly include fever, headache, fatigue, and pain at the injection site [5]. More severe but rare side effects include anaphylaxis [6] and thromboembolic events [7]. A prospective observational study in the United Kingdom examined self-reported systemic (eg, headache and fever) and local (eg, pain and redness at the injection site) side effects. It found that side effects in the community were less prevalent than what was expected from phase 3 clinical trials [2]. They were also more prevalent in females than males, in younger individuals compared to older individuals, and in individuals with a higher BMI compared to those with a lower BMI.

Additionally, side effects in the study by Menni et al [2] were more common in individuals who had had a previous SARS-CoV-2 infection compared to their infection-free peers. The authors proposed that the higher reactogenicity in previously infected individuals may be linked to higher immunogenicity leading to higher antibody titers as previously observed [8]. Postvaccination physical symptoms arise as manifestations of inflammatory events mimicking the response to a natural infection [9]. Such inflammatory events are important for the development of protection against the vaccine-targeted disease.

Whether triggered by a vaccine or the disease itself, inflammatory immune responses are often detectable via changes in physiological signals (eg, high fever). Risch et al [10] documented an increase in heart rate, breathing rate, and wrist skin temperature as well as a decrease in heart rate variability during a SARS-CoV-2 infection. Their findings underscore a broader shift toward personalized medicine, with recent systematic reviews demonstrating how monitoring physiological signals can serve as an important tool in digital health care [11]. In particular, modern wearable technology offers an opportunity to collect real-time physiological data remotely, reliably and continuously in order to inform medical decisions. Recently, physiological signals have been suggested as a proxy for vaccine reactogenicity [12]. In response to COVID-19 vaccination, a short-term increase in heart rate has consistently been measured by means of wearable technology [12-15]. Several confounding factors have been identified including age, vaccine type, and prior SARS-CoV-2 infection [12]. Furthermore, differences between female and male physiological reaction to vaccination have been reported [12], highlighting the importance of considering sex as a biological variable in vaccine-induced immune response.

Further supporting sex-based analyses, physiological signals are strongly impacted by hormones and the menstrual cycle [16-18]. For example, shifts in wrist-skin temperature, heart rate, and breathing rate have been demonstrated and tracked by wearable devices across the menstrual cycle [16]. Accordingly, sex differences in the physiological response to vaccines are important to take into account. Previously, female participants were often underrepresented or entirely excluded in clinical trials, leading to a lack of evidence regarding the response to specific drugs and treatments in female patients [19]. However, different immune responses to non–COVID-19 vaccines have been observed between female and male participants including higher antibody responses and more reported adverse events in female participants [20]. Therefore, it is crucial to stratify reports on vaccine biology by sex [21].

Furthermore, anecdotal reports of COVID-19 vaccine effects on the menstrual cycle have led to discussions in the media and caused concerns among the general public [22,23]. Thus far, only small, short-term decreases have been observed in self-reported menstrual cycle duration following vaccination [24,25]. Moreover, among 39,129 respondents to a web-based survey, increased bleeding after vaccination was reported by 42% of people with regular menstrual cycles [26]. However, such changes in menstrual bleeding intensity do not necessarily indicate changes in fertility following vaccination [27]. Similarly, contrary to initial public concern, COVID-19 mRNA vaccination led to *increases* in sperm concentration and motility as well as semen volume [28]. In contrast, a SARS-CoV-2 infection in male participants may be associated with a short-term decline in fertility [27], thereby suggesting vaccinations may have a potential protective factor for male fertility.

In this study, we sought to quantify the physiological immune response to COVID-19 vaccination by looking for sex-based differences in wearable-measured physiological parameters. We investigated temporal dynamics of 4 physiological parameters: breathing rate, wrist skin temperature, heart rate, and heart rate variability before and after COVID-19 vaccination. Further focusing on sex-specific changes, we objectively assessed the duration of different phases of the menstrual cycle before and after vaccination. Since information about potential vaccine-induced changes to specific menstrual cycle phases is lacking and discrepancies in these biologically distinct periods may affect the interpretation of vaccination effects, this is an important knowledge gap to fill.

## Methods

### Study Design

In a prospective trial, the COVID-19 Remote Early Detection (COVID-RED) trial [29], we investigated the use of a wrist-worn device for remote early detection of SARS-CoV-2 infections. The trial has been registered at the Netherlands Trial Register (see [29] for details and the study protocol). The study’s main objectives aimed to detect infection-based deviations in the measured physiological parameters from their baseline in both male and female participants; the current analysis constitutes an exploratory objective of the main COVID-RED clinical trial.

Over the course of the COVID-RED trial (from February to November 2021), 17,825 participants from the Netherlands wore the CE-certified and Food and Drug Administration–cleared Ava Fertility Tracker (Ava AG) for up to 9 months. Originally built to detect a woman’s fertile days in real time, the wearable bracelet tracks biophysical changes in heart rate, breathing rate, skin temperature, and heart rate variability with 3 types of sensors: a photoplethysmograph, an accelerometer and a temperature sensor. In this study, sensor data were recorded every 10 seconds during the user’s sleep and synchronized with a complementary smartphone app (“Ava COVID-RED”) upon waking. The COVID-RED study procedure has been previously published [29]. Briefly, in addition to wearing the Ava bracelet nightly, participants entered information about their daily health status and behavior including experience of any symptoms and vaccinations in the Ava COVID-RED app. Furthermore, they filled out biweekly surveys allowing us to collect information on health care use, COVID-19 testing, behavioral changes, and COVID-19 vaccination. Finally, they collected a capillary blood sample upon enrolment and every 3 months throughout the trial allowing for a laboratory confirmation of a SARS-CoV-2 infection. Based on these data, we examined the trajectory of physiological parameters in a subset of participants who reported at least one COVID-19 vaccination event across the two sources (ie, Ava COVID-RED app and the biweekly survey).

### Statistical Analysis

Only participants with successful synchronizations of bracelet data with the Ava COVID-RED app on 15 of 20 days before as well as after their first COVID-19 vaccination were included in the current analyses. All participants who reported more than one vaccination event within this period (ie, 20 days before and 20 days after the first COVID-19 vaccination) were excluded to facilitate analysis of specific effects associated with the first dose of the COVID-19 vaccine.

### Part 1: Physiological Signals Around Vaccination

With a total of 2189 participants from 19 to 80 years old (mean 50.32, SD 13.17 years; 1613 female; see Table 1), regression models were constructed to examine the dynamics of 4 physiological parameters around vaccination (ie, breathing rate in breaths per minute, skin temperature in degree Celsius, heart rate in beats per minute, and heart rate variability). Heart rate variability was quantified as the ratio of heart rate oscillations with low frequency (0.04-0.15 Hz) to those with high frequency (0.15-0.4 Hz), as described by Risch et al [10]. For each of the 4 parameters, 4 models were built and compared with regard to their Akaike Information Criterion, conditional *R*^2^, and root-mean-square error with daily parameter measurements as the outcome variable. The four models included: (1) only a random intercept; (2) sex, menstruation status, age, BMI, type of vaccine (ie, Pfizer-BioNTech [BNT162], Moderna [mRNA-1273], AstraZeneca [AZD1222], and Janssen [Ad26.COV2.S]), existence of laboratory-confirmed SARS-CoV-2 infection prior to vaccination, and sleep duration as fixed factors and number of days from vaccination as a fixed quadratic term; (3) random intercept in addition to the fixed factors from model 2; and (4) interaction of the quadratic term with sex, existence of SARS-CoV-2 infection prior to vaccination, and sleep duration in addition to all terms from model 3. We did not expect a linear change in physiological parameters in response to vaccination; rather, we sought to model a quadratic effect of vaccination-induced changes reflected in a short-term alteration in biophysical parameters before a return to prior baseline values. For this term, we quantified the time from vaccination in number of days with 0 reflecting the day of vaccination, negative values reflecting days preceding vaccination and positive values reflecting days following vaccination. This method adequately models the initial impact and subsequent adjustment postvaccination, better reflecting the complex temporal dynamics than linear models. In line with standard approaches to time-varying effect sizes [30], it reflects the differential impact of COVID-19 vaccination on biophysical parameters before their return to baseline. The quadratic multilevel model provides a nuanced analysis of physiological responses over time, justifying its selection over simpler approaches for our longitudinal data. For models 2-4, we categorized female participant’s menstruation status into 3 categories: not menstruating (including both pregnant and postmenopausal participants), perimenopausal, and menstruating. Interaction terms were included in model 4 to capture potential differences in factors previously identified as potentially influencing COVID-19 vaccine reactogenicity [12].

**Table 1:** Demographic characteristics stratified by vaccine type.

Characteristics		All COVID-19 vaccines		Pfizer-BioNTech (BNT162)			Moderna (mRNA-1273)			AstraZeneca (AZD1222)			Janssen (Ad26.COV2.S)	
		Female (n=1613)	Male (n=576)	Female (n=1142)	Male (n=389)	Female (n=221)		Male (n=78)	Female (n=153)		Male (n=62)	Female (n=28)		Male (n=16)
Age (years), mean (SD)		49.32 (12.8)	52.71 (13.86)	48.6 (12.85)	52.53 (14.5)	44.34 (10.54)		45.27 (9.84)	59.61 (99.9)		61.32 (8.7)	49.5 (10.28)		46.07 (13.05)
BMI (kg/m^2^), mean (SD)		26.88 (5.44)	26.27 (4.17)	26.95 (5.46)	26.46 (4.25)	25.76 (4.45)		25.85 (4.38)	27.75 (6.32)		26.55 (3.52)	26.37 (4.91)		24.93 (4.23)
**Menstruation status, n**														
	Not menstruating	223	N/A^a^	169	N/A	27		N/A	17		N/A	4		N/A
	Pregnant	2	N/A	2	N/A	0		N/A	0		N/A	0		N/A
	Premenopausal	642	N/A	480	N/A	119		N/A	17		N/A	11		N/A
	Perimenopausal	168	N/A	122	N/A	28		N/A	6		N/A	2		N/A
	Postmenopausal	577	N/A	368	N/A	47		N/A	113		N/A	11		N/A

^a^N/A: not applicable.

### Part 2: Menstrual Cycle Duration Around Vaccination

To investigate the differences in menstrual cycle duration following COVID-19 vaccination, phases of the menstrual cycle were determined based on fluctuations in physiological signals and participant-reported menses by means of a proprietary algorithm (Ava AG). Of note, the algorithm’s effectiveness is not solely dependent on the precise reporting of the cycle’s onset but rather on its ability to learn by aggregating and analyzing biophysical data points across women’s repeated menstrual cycles (as captured during the 9-month COVID-RED study). Ava’s fertility detection algorithm has been previously validated in clinical trials, with its accuracy on par with urine-based ovulation tests [16,31]. By means of this method, the following phases were detected: menstrual phase (ie, 5 days from the first day of menses), follicular phase (ie, from the first day post menses through 6 days before ovulation), fertile window (ie, 5 days before ovulation and the ovulation day), early luteal phase (ie, from the first day after ovulation through a week after ovulation), and late luteal phase (ie, from the eighth day after ovulation through the day before the onset of subsequent menses). As these algorithms’ performance depends on the reliability of the physiological data, we included only those female participants with synchronized data for each day of each menstrual cycle and more than 1 available cycle (n=179 from 19 to 55 years of age; mean age 41.31, SD 8 years). For this analysis, a variable describing the duration of each cycle phase in the current cycle was created to serve as the outcome variable in regression models. As in part 1, for each of the 5 phases, we applied four models including: (1) only a random intercept; (2) age, BMI, daily alcohol use, contraceptive method, type of COVID-19 vaccine, existence of laboratory-confirmed SARS-CoV-2 infection prior to vaccination and sleep duration as fixed factors and number of days from vaccination as a fixed quadratic term; (3) random intercept in addition to the fixed factors from model 2; and (4) interaction of the quadratic term with existence of SARS-CoV-2 infection prior to vaccination and sleep duration in addition to all terms from model 3. In addition to information about COVID-19 vaccination and infection, only factors that have previously been shown to significantly impact menstrual cycle duration were included [32-34].

For all analyses, the reported *P* values were corrected by means of the false discovery rate [35] to maintain a family-wise α rate of .05 across the performed statistical tests (ie, the number of calculated *P* values in the 9 models examining the 4 physiological parameters and the 5 phases of the menstrual cycle as the outcome).

### Ethical Considerations

The study was approved by the responsible ethics committee Medisch Ethische Toetsingscommissie Utrecht (SL/nb/21/500101), and electronic informed consent was provided by each participant. Participants were informed that their participation in the study is voluntary and that they could withdraw from the study at any time. No compensation was offered for participation. Participant data were pseudonymized immediately after collection to ensure confidentiality and privacy.

## Results

### Population characteristics

The 2189 included participants reported receiving 4 different types of COVID-19 vaccines (Table 1). The majority (1531/2189, 70%) received the vaccine from Pfizer-BioNTech (BNT162), followed by 14% (299/2189) reporting vaccination with Moderna (mRNA-1273), 10% (215/2189) AstraZeneca (AZD1222), and 2% (44/2189) Janssen (Ad26.COV2.S).

### Part 1: Physiological Signals Around Vaccination

**Figure 1:**
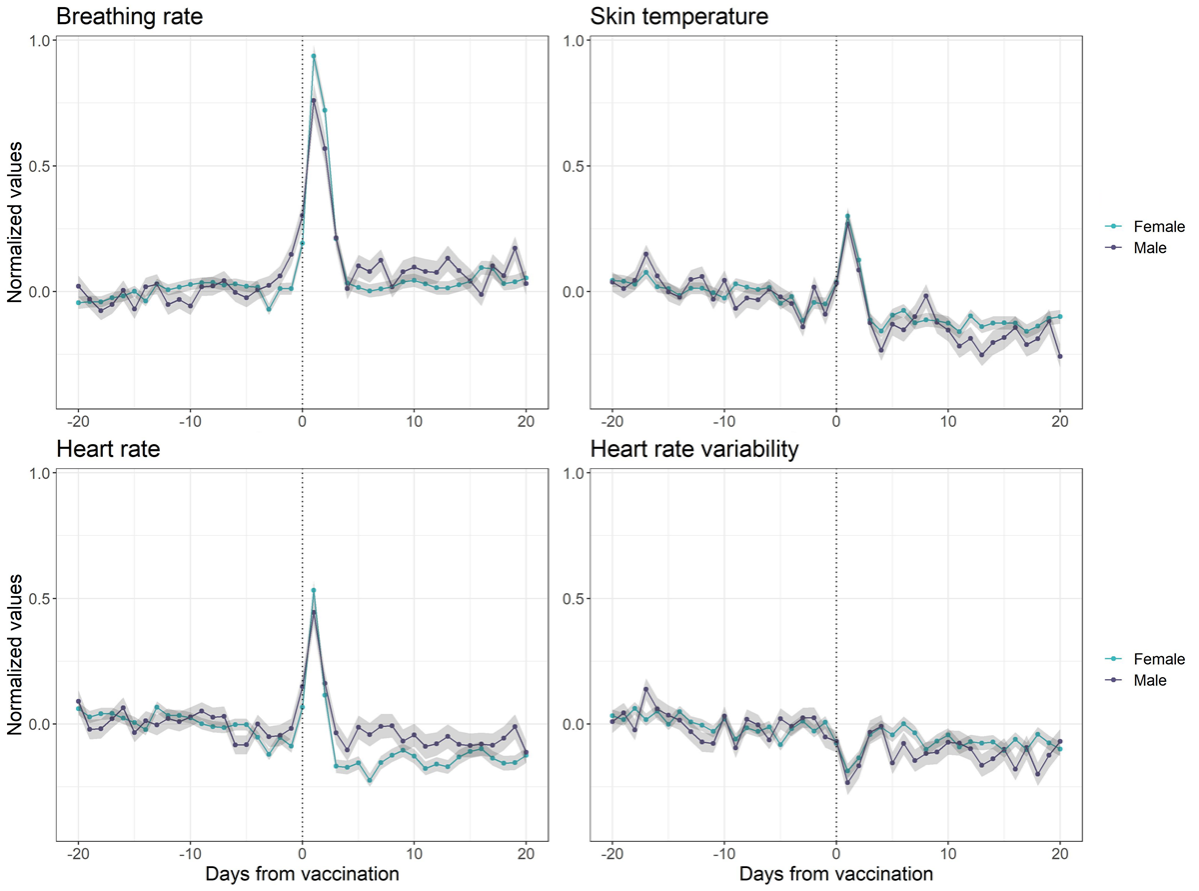
Trajectory of physiological signals around COVID-19 vaccination. The values of each parameter (with standard deviation in gray) were normalized according to each individual’s mean baseline measurement (ie, during days before vaccination) and averaged across female (n=1613) and male participants separately (n=576).

The trajectory of the 4 physiological parameters across 2189 participants showed short-term changes in the first days after vaccination and a rebound to baseline levels measured before vaccination thereafter (Figure 1). Accuracy metrics of the 4 models for each parameter are shown in Table S1 in Multimedia Appendix 1 and indicate the models including fixed factors and a random intercept as most suitable (ie, model 3). The only exception was observed for heart rate variability with the model additionally including interactions between factors showing the best accuracy (ie, model 4). From this point onward, we will exclusively focus on the results obtained from the best fitting model described above. The short-term increase in breathing rate and heart rate was revealed as statistically significant. This increase was reflected by a negative effect of the quadratic term depicting days since vaccination, indicating that breathing rate and heart rate initially rise but then decrease again as time moves away from the vaccination event (Table 2). Nevertheless, the interaction between sex and days since vaccination remained nonsignificant (Table 2), suggesting no meaningful sex differences in the physiological response to COVID-19 vaccination. Similarly, the nonsignificant interaction with sleep duration (Table 2) indicates no impact of sleep duration on the physiological response to vaccination. Finally, the qualitative short-term decrease in heart rate variability following vaccination was not apparent in individuals who experienced a SARS-CoV-2 infection prior to vaccination (interaction effect in Table 2; Figure S1 in Multimedia Appendix 1).

Regardless of the vaccination event, female participants demonstrated higher wrist skin temperature and heart rate as well as a lower heart rate variability than male participants (Table 2). Furthermore, older participants showed a lower breathing rate as well as a higher heart rate variability as reflected in significant effects of age (Table 2). Participants with a higher BMI exhibited higher breathing rate and heart rate but lower skin temperature and heart rate variability (Table 2). Perimenopausal and menstruating female participants had a higher skin temperature, while menstruating female participants also had a higher breathing rate than nonmenstruating participants (Table 2). Finally, longer sleep duration was associated with lower breathing rate and heart rate as well as higher skin temperature and heart rate variability (Table 2). There were no significant effects of vaccine type (Table 2).

**Table 2:** Results from multilevel quadratic regression models including physiological signals from 2189 participants (1613 females) around COVID-19 vaccination^a^.

		Breathing rate (breaths/min)	Skin temperature (°C)	Heart rate (beats/min)	Heart rate variability
Intercept		*13.41 (<.0001)*	*36.1 (<.0001)*	*43.8 (<.0001)*	*3 (<.0001)*
**Main effects**					
	Days from vaccination	–*0.04 (<.0001)*	–0.004 (.36)	–*0.03 (.04)*	–0.001 (1)
**Vaccine type**					
	AstraZeneca	Reference	Reference	Reference	Reference
	Janssen	0.11 (1)	0.12 (.92)	–0.49 (1)	–0.11 (1)
	Moderna	0.08 (1)	0.07 (.58)	0.54 (1)	0.07 (1)
	Pfizer-BioNTech	0.39 (.19)	0.07 (.36)	0.43 (1)	0.04 (1)
Previous infection		0.56 (.73)	–0.003 (1)	0.02 (1)	0.11 (1)
Sex		0.3 (.19)	*0.18 (<.0001)*	*3.1 (<.0001)*	–*0.72 (<.0001)*
Age		–*0.02 (<.0001)*	–0.002 (.36)	0.03 (.11)	*0.007 (.03)*
BMI		*0.08 (<.0001)*	–*0.02 (<.0001)*	*0.32 (<.0001)*	–*0.04 (<.0001)*
**Menstruation status**					
	Not menstruating	Reference	Reference	Reference	Reference
	Perimenopausal	0.37 (.65)	*0.12 (.02)*	0.61 (1)	0.02 (1)
	Menstruating	*0.44 (.02)*	*0.16 (<.0001)*	0.76 (1)	–0.05 (1)
Sleep duration		–*0.06 (<.0001)*	*0.04 (<.0001)*	–*0.46* *(<.0001)*	*0.01 (.004)*
**Interactions**					
	Sex×Days from vaccination	N/A^b^	N/A	N/A	0.008 (.98)
	Previous infection×Days from vaccination	N/A	N/A	N/A	–*0.07 (<.0001)*
	Sleep duration×Days from vaccination	N/A	N/A	N/A	–0.004 (.87)

^a^Unstandardized β coefficients are presented with *P* values in parentheses and significant effects italicized (*P*<.05). Sex was coded such that positive coefficients represent greater values in females.

^b^N/A: not applicable.

### Part 2: Menstrual Cycle Duration Around Vaccination

**Figure 2:**
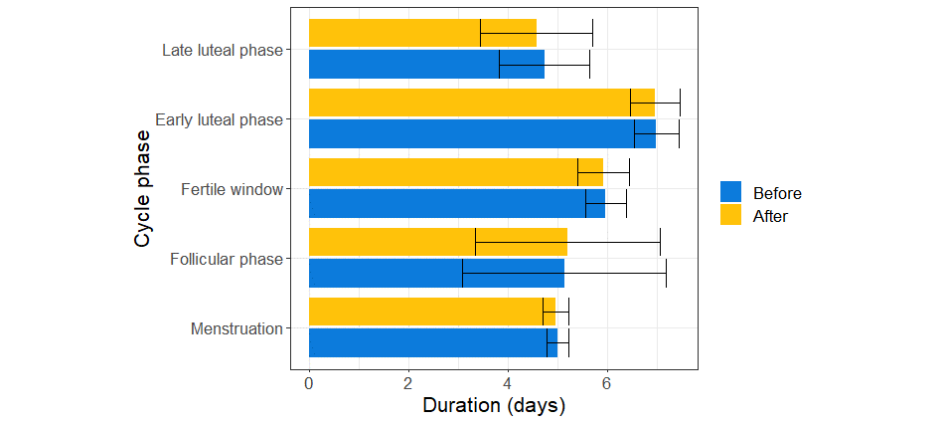
Mean duration of each cycle phase in days before and after vaccination across 179 female participants with 2 to 8 cycles per subject (mean number of cycles included in analysis 3.59, SD 1.36). The error bars depict the standard deviation.

Our analysis of menstrual cycle duration revealed a significant positive effect of the quadratic term representing time since vaccination on menstrual phase duration. This finding suggests a decrease in menstrual phase duration around the time of vaccination. In contrast, we found a trend-level increase in duration of follicular phase following vaccination (Table 3). The mean within-participant difference between pre- and postvaccine duration of the menstrual cycle revealed that this change amounted to 0.8% for the menstrual phase and 1.4% for the follicular phase (Figure 2). We found an overall decrease in total cycle duration of 0.26 days following vaccination (Table S2 in Multimedia Appendix 1). Accuracy metrics of the 4 models for each cycle phase are shown in Table S3 in Multimedia Appendix 1 and indicate the models including fixed factors and a random intercept as most suitable (ie, model 3).

Regardless of the vaccination event, older females experienced a shorter follicular phase (Table 3). Furthermore, alcohol use was associated with a shorter fertile window (Table 3). There were no significant effects of vaccine type, contraceptive method, or BMI on menstrual cycle duration (Table 3).

**Table 3:** Results from multilevel quadratic regression models including 642 cycles from 179 female participants who received a COVID-19 vaccine^a^.

		Menstrual phase duration	Follicular phase duration	Fertile window duration	Early luteal phase duration	Late luteal phase duration
Intercept		*4.81 (<.0001)*	*6.22 (.02)*	*5.89 (<.0001)*	*7.01 (<.0001)*	*4.04 (.002)*
**Main effects**						
	Days from vaccination	*0.007 (.0003)*	–0.04 (.06)	0.005 (1)	0.009 (.31)	–0.008 (1)
**Vaccine type**						
	AstraZeneca	Reference	Reference	Reference	Reference	Reference
	Janssen	0.12 (1)	0.17 (1)	–0.65 (1)	0.5 (1)	–1.24 (1)
	Moderna	0.22 (1)	0.69 (1)	–0.41 (1)	0.13 (1)	–0.67 (1)
	Pfizer-BioNTech	0.21 (1)	0.25 (1)	–0.22 (1)	0.25 (1)	–0.75 (1)
Previous infection		0.4 (1)	3.16 (1)	–0.19 (1)	–0.04 (1)	1.01 (1)
Age		0.003 (1)	–*0.1 (.02)*	0.01 (.8)	–0.002 (1)	0.02 (1)
BMI		–0.006 (1)	0.11 (.38)	–0.009 (1)	–0.003 (1)	0.02 (1)
**Contraceptive method**						
	None	Reference	Reference	Reference	Reference	Reference
	Hormonal	0.41 (1)	0.07 (1)	0.16 (1)	–0.13 (1)	–0.63 (1)
	Intrauterine device	0.13 (1)	–0.36 (1)	–0.26 (1)	–0.05 (1)	–0.23 (1)
	Other	0.02 (1)	0.81 (1)	0.05 (1)	–0.1 (1)	–0.13 (1)
**Alcohol use**						
	None	Reference	Reference	Reference	Reference	Reference
	1-2 drinks	–0.002 (1)	0.005 (1)	–*0.05 (<.0001)*	0.002 (1)	–0.04 (.38)
	3-4 drinks	–0.008 (1)	–0.02 (1)	–*0.07 (.02)*	0.02 (1)	–0.1 (.1)
	5+ drinks	–0.001 (1)	0.08 (1)	0.01 (1)	–0.07 (.31)	–0.05 (1)
Sleep duration		–0.003 (1)	0.006 (1)	–0.004 (1)	0.004 (1)	–0.005 (1)

^a^Unstandardized β coefficients are presented with *P* values in parentheses and significant effects italicized (*P*<.05).

## Discussion

### Principal Findings

This study examined the effects of COVID-19 vaccination on several physiological signals measured continuously for up to 9 months in 2189 adult participants. We found short-term increases in breathing rate and heart rate following vaccination, which did not differ between the sexes. Furthermore, we observed a 0.8% decrease in menstrual phase duration after vaccination as compared to before. Taken together, our findings indicate that COVID-19 vaccines may cause physiological reactions in line with other routine vaccines [36,37] that typically last only a few days.

### Comparison With Prior Work

The observed short-term physiological response parallels the duration of self-reported side effects from COVID-19 vaccination in the community [2] as well as previous reports of physiological response to COVID-19 vaccination [12-14]. During the immune reaction to any vaccination, inflammatory responses may be propagated to the central nervous system causing systemic side effects such as fever and headache [9]. The activated neuronal circuits then activate autonomic responses like peripheral vasoconstriction [9]. Such modulations may cause the observed physiological alterations following vaccination in our study. For example, peripheral vasoconstriction is associated with increases in heart rate [38], which is in turn positively associated with breathing rate [39], supporting our findings.

Additionally, decreased heart rate variability during inflammatory response to natural infection [40] as well as after influenza vaccination [41] has been observed in electrocardiographic measurements. Such observations are not surprising, given that heart rate variability reflects vagal and thereby autonomic nervous system activity [42]. Although, on average, we observed decreases in heart rate variability in the first days after vaccination, these changes did not reach statistical significance. This observation is in line with a previous study on wearable-measured physiological signals during the course of SARS-CoV-2 infection, which reported less pronounced changes in heart rate variability as compared to heart rate and breathing rate [10]. Similarly, while skin temperature did increase in response to vaccination, this increase was not significant in our study. This finding is consistent with the report by Gepner et al [13], showing a relative change of below 1% for skin temperature and above 5% for heart and respiratory rate following COVID-19 vaccination.

We observed no differences between the sexes in the physiological response to vaccination. For the Pfizer-BioNTech and Moderna vaccines, slightly lower (2.3%-2.7%) efficacy has been reported in female participants compared to male participants [43,44]. In contrast, the efficacy of non–COVID-19 routine vaccines in the older participants is greater in female participants than in male participants [45], suggesting an important relation with age [21]. Genetics constitute an additional factor for consideration, as highlighted by twin studies that show a heritability of 36% to 90% for immune response to vaccines [46]. Importantly, a larger increase in breathing rate has recently been measured in female participants as compared to male participants after COVID-19 vaccination [12]. Although our findings appear to suggest a larger initial physiological response to vaccination in female participants for breathing rate, heart rate, and skin temperature (Figure 1), this difference is small and did not reach statistical significance. As the lack of statistical significance does not necessarily imply a lack of clinical significance [47], we recommend that future studies specifically examine the clinical relevance of sex differences in the context of vaccine reactogenicity. Interactions among genetic factors, age, and sex hormones render the biological mechanisms behind sex differences complex, potentially masking the sex effects in our sample. Nonetheless, such effects are important to consider and report in studies to ensure that the needs of male and female subgroups have been adequately addressed.

In addition to sex differences, the COVID-RED data set allowed us to examine changes in duration of different menstrual cycle phases following vaccination. We found that the follicular phase slightly increased in duration, while the menstrual phase was shorter after vaccination. Such dynamics could obscure vaccination effects on the overall duration of the menstrual cycle. Prior work found that the menstrual cycle was less than 1 day longer immediately after as compared to before vaccination [24]. The overall decrease in menstrual cycle duration in our study contradicts this previous finding. The discrepancy may be due to methodological differences, as Edelman et al [24] examined self-reported durations, while we additionally relied on objective fluctuations in physiological signals to determine cycle phases using machine learning. Despite this difference, both investigations showed a small change of less than 1 day in menstrual cycle duration following vaccination. Similarly, irregularities in the menstrual cycle have been reported after non–COVID-19 vaccines including missed, late, and early menstrual bleeding [48,49]. These findings hint at large interindividual differences among menstruating women in response to vaccination, which are also reflected in the wide variance observed in the duration of specific menstrual cycle phases depicted in Figure 2. The mechanisms behind such observations may be linked to alterations in mast cell activation, which is implicated in both inflammatory processes [50] as well as the initiation of menstruation [51]. Further factors known to influence these interactions also likely contribute to interindividual variability and inconsistency between findings. For instance, age at onset of menarche and adiposity have been shown to affect the relationship between inflammatory biomarkers and ovarian function [52,53]. Taken together, we cannot exclude the possibility that vaccination may cause small temporal shifts of the fertile window in some female participants with consequences for their chances to conceive. Finally, as our study design did not allow for an investigation of long-term effects of COVID-19 vaccination on menstrual cycle duration, we cannot draw any conclusions about the long-term regularity of menstrual cycles postvaccination. It is, however, important to note that previous research has reported links between menstrual cycle irregularities and increased risk of cardiovascular disease [54] as well as diabetes [55]. This underscores the necessity for future research to examine vaccine-induced changes in menstrual cycle characteristics over longer periods of time in order to understand their potential clinical implications.

Across analyses, the 4 examined types of COVID-19 vaccines did not differ with regard to the observed physiological responses. The COVID-19 vaccines by Pfizer-BioNTech and Moderna use the mRNA isolated from the SARS-CoV-2 spike protein to induce synthesis of this protein in host cells upon injection and initiate an immune response [56]. The AstraZeneca vaccine uses a modified chimpanzee DNA adenovirus that does not cause an immune response to the virus but only to the SARS-CoV-2 spike protein produced in host cells after injection [5,57]. Finally, the vaccine developed by Janssen uses an inactivated adenovirus vector that codes for the viral spike protein [57] and thus induces a similar host response as the AstraZeneca vaccine. Regardless of their type, all COVID-19 vaccines ultimately activate the same immune pathways in the host and were similarly reflected in physiological responses measured in this study.

### Limitations

Several limitations are important to acknowledge in the context of this study. First, the self-reported nature of the data collected through the Ava COVID-RED app and the biweekly surveys may introduce subjective bias into our findings as they rely on participants’ recall. To mitigate this potential risk, we strategically designed the Ava COVID-RED app to ask for daily input of self-reported data. Additionally, participants could only edit their responses for up to a week. Furthermore, we collected information in a redundant manner through multiple sources whenever possible. Therefore, we believe that the key measures of our study (ie, information about menstrual cycle start and COVID-19 vaccination) are reliable. Further, we recognize that there may be additional factors arising from pandemic-induced changes in lifestyle and particularly stress levels that could affect physiological signals. However, the observed effects are specific to the immediate postvaccination period and rely on relative changes in physiological signals from each individual’s baseline (measured during the pandemic), while the trajectory of physiological signals remains consistent with baseline levels prior to vaccination. Therefore, we argue that these contextual factors as well as potential acute stress or anxiety anticipating vaccination are unlikely to confound our findings significantly. Moreover, while we acknowledge variability in wearable device accuracy [58,59], the large sample size and the temporal specificity of the observed effects mitigate this concern. Finally, this study’s cohort was recruited exclusively from the Netherlands, potentially limiting the generalizability of our findings to other countries and thus calling for replication across diverse populations.

This study examined the physiological response to the first dose of COVID-19 vaccine. Most participants received their first dose during the last trimester of the trial, yielding only a small sample with sufficient data to investigate the trajectory of physiological signals after the second dose. We thus refrained from conducting this additional analysis due to lack of statistical power and recommend that future studies investigate potential impact of further doses as well as long-term effects thereof. It is imperative that such studies continue to consider potential sex-specific responses to vaccination, particularly concerning menstrual cycle changes, and assess the clinical relevance of the minor and transient physiological changes observed in our study. Understanding these differences is vital for tailoring public health policies and recommendations for vaccine development to better serve all segments of the society. Finally, the continuous, real-time data provided by wearable technology offer an unprecedented opportunity to advance such efforts, facilitating a move toward more personalized health care.

### Conclusions

This study constitutes the first comprehensive examination of continuous measurements of multiple physiological signals as well as objectively assessed menstrual cycle duration around a vaccination event. We rely on data collected by a wearable medical device in a large-scale clinical trial including contextual information such as the type of COVID-19 vaccine received to ensure higher validity results and find short-term changes in breathing rate and heart rate following vaccination. The prompt rebound to baseline levels measured before vaccination suggests that the COVID-19 vaccines are not associated with any long-term effects on heart rate, breathing rate, and heart rate variability or skin temperature. Similarly, our investigation of changes in the menstrual cycle duration showed minimal deviations following vaccination for several phases of the cycle. Based on these observations, we recommend that female participants relying on the regularity of their menstrual cycle both to prevent conception or conceive be particularly cautious during their menstrual cycles immediately following vaccination. Future investigations should consider whether this phenomenon extends to non–COVID-19 vaccines. We emphasize the importance of ongoing monitoring and further research to comprehensively assess the long-term impacts of these vaccines on physiological parameters and the menstrual cycle. Such extended research is vital to address remaining uncertainties and reinforce confidence in vaccine safety through robust, longitudinal data analysis, leveraging the demonstrated potential of wearable technology. Taken together, our work provides valuable insights into continuous fluctuations of our body’s responses to vaccination and may help refute misinformation about vaccines contributing to fertility or biophysical issues.

## Appendix

Multimedia Appendix 1 Accuracy metrics of statistical models; numbers of reported vaccinations for analysis of menstrual cycle duration changes; and interaction between the number of days from vaccination and previous infection in influencing the trajectory of heart rate variability.

## Abbreviations

COVID-RED: COVID-19 Remote Early Detection

## Data Availability

The data sets generated and analyzed during this study are available in the DataverseNL repository [63].
