# Supplementary material for: Physiological Response to the COVID-19 Vaccine: Insights From a Prospective, Randomized, Single-Blinded, Crossover Trial
Source: J Med Internet Res. 2024 Jul 31;26:e51120. doi: 10.2196/51120 (PMC11325110; doi:10.2196/51120)

This document presents the accuracy metrics of all statistical models calculated in the study. Additionally, it includes the numbers of reported vaccinations for our analysis of menstrual cycle duration changes. Finally, we provide a visualization illustrating the interaction between the number of days from vaccination and previous infection in influencing the trajectory of heart rate variability.

Table S1. Accuracy metrics (Akaike Information Criterion [AIC], conditional R^2 and root mean squared error [RMSE]) for each of the evaluated models and the four parameters. The model with the lowest AIC and RMSE as well as the highest R^2 was selected and used for further analysis (in bold).

		Breathing rate	Skin temperature	Heart rate	Heart rate variability
Model 1) Intercept-only	AIC	230780.1	107732.8	442228.5	147849
	R^2	0.87	0.51	0.84	0.82
	RMSE	0.86	0.43	3	0.53
Model 2) Fixed factors	AIC	230164.1	106969	440679.6	147637.9
	R^2	0.87	0.51	0.84	0.82
	RMSE	0.86	0.43	2.98	0.53
Model 3) Random factors	AIC	**228072.5**	**106137.4**	**437486.8**	144840.5
	R^2	**0.87**	**0.52**	**0.85**	0.83
	RMSE	**0.83**	**0.42**	**2.84**	0.51
Model 4) Interactions	AIC	228094.6	106169.5	437493.1	**144839.8**
	R^2	0.87	0.52	0.85	**0.83**
	RMSE	0.83	0.42	2.84	**0.51**

Table S2. Number of reported vaccinations occurring during a given phase of the menstrual cycle for each vaccine type. The average within-subject difference between pre- and post-vaccination duration were calculated for each cycle phase. A total of 642 cycles from 179 females who received a COVID-19 vaccine were included.

	Menstrual phase	Follicular phase	Fertile window	Early luteal phase	Late luteal phase
Number of vaccinations					
AstraZeneca (AZD1222)	2	4	1	2	0
Janssen (Ad26.COV2.S)	2	2	0	1	0
Moderna (mRNA-1273)	7	5	4	10	1
Pfizer/BioNTech (BNT162)	25	22	31	24	16
Difference in duration from before to after vaccination (mean ± SD in days)	0.04 ± 0.33	-0.04 ± 2.07	0.05 ± 0.64	0.04 ± 0.65	0.17 ± 1.25

Table S3. Accuracy metrics (Akaike Information Criterion [AIC], conditional R^2 and root mean squared error [RMSE]) for each of the evaluated models and the five phases of the menstrual cycle. The model with the lowest AIC and RMSE as well as the highest R^2 was selected and used for further analysis (in bold).

		Menstrual phase	Follicular phase	Fertile window	Early luteal phase	Late luteal phase
Model 1) Intercept-only	AIC	-8656.81	49396.11	21318.07	18274.28	37567.89
	R^2	0.56	0.56	0.37	0.4	0.45
	RMSE	0.17	1.3	0.49	0.44	0.82
Model 2) Fixed factors	AIC	-8575.62	49395.04	21354.96	18324.7	37622.8
	R^2	0.57	0.56	0.38	0.41	0.46
	RMSE	0.17	1.3	0.49	0.44	0.82
Model 3) Random factors	AIC	**-16294.01**	**42181.29**	**16734.92**	**8032.33**	**30858.29**
	R^2	**0.9**	**0.9**	**0.72**	**0.86**	**0.86**
	RMSE	**0.13**	**0.97**	**0.4**	**0.29**	**0.62**
Model 4) Interactions	AIC	-16277.27	42188.11	16751.46	8048.58	30867.28
	R^2	0.9	0.9	0.72	0.86	0.86
	RMSE	0.13	0.97	0.4	0.29	0.62

Figure S1. Trajectory of heart rate variability around COVID-19 vaccination. The values (with standard deviation in grey) were normalized according to each individual's mean baseline measurement (i.e., during days before vaccination) and averaged across individuals with a COVID-19 infection (n=54) and those without a COVID-19 infection (n=1000) prior to vaccination.

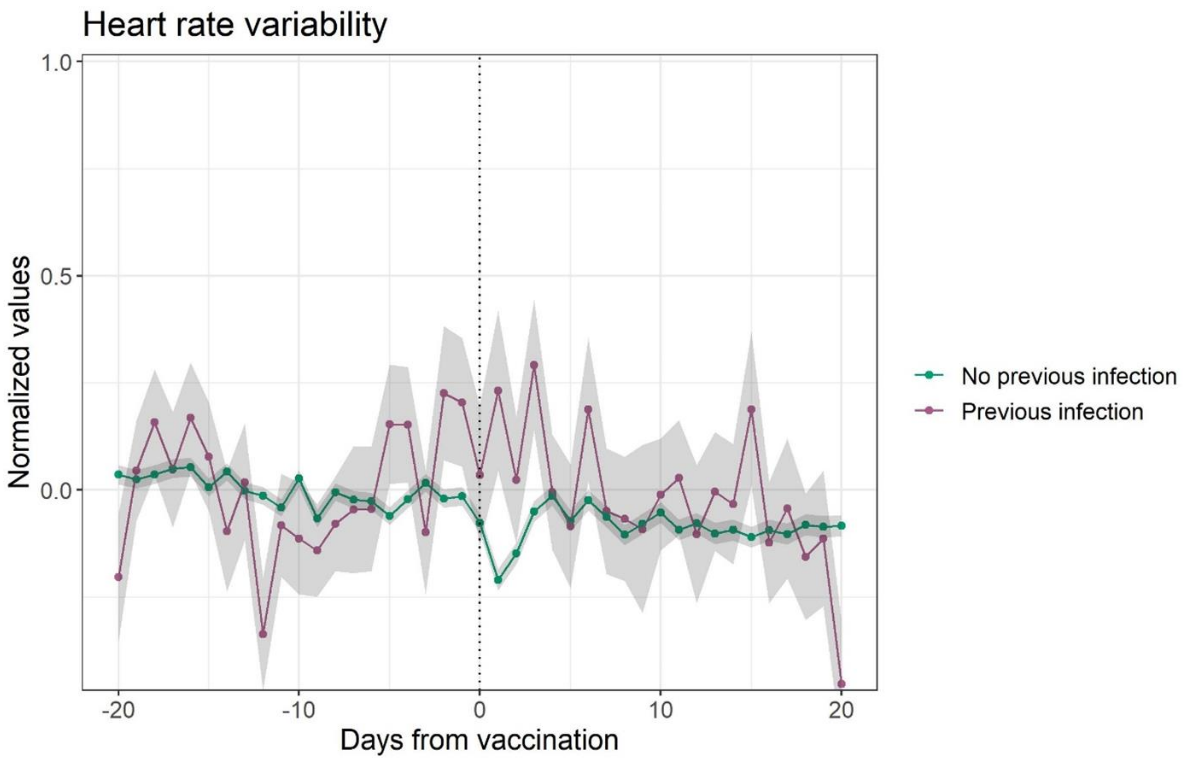

Supplement: Multimedia Appendix 1 [file jmir_v26i1e51120_app1.pdf]
